# PDZ Domains and Viral Infection: Versatile Potentials of HPV-PDZ Interactions in relation to Malignancy

**DOI:** 10.1155/2013/369712

**Published:** 2013-09-04

**Authors:** Kazunori Nagasaka, Kei Kawana, Yutaka Osuga, Tomoyuki Fujii

**Affiliations:** Department of Obstetrics and Gynecology, Faculty of Medicine, The University of Tokyo, Tokyo 113-8655, Japan

## Abstract

Cervical cancer is caused by high-risk human papillomaviruses (HPVs), and a unique characteristic of these is a PDZ ($\underline{\text{P}}$SD-95/$\underline{\text{D}}$lg/$\underline{\text{Z}}$O-1-)binding motif in their E6 proteins. Through this motif HPV E6 interacts with a variety of PDZ domain-containing proteins and targets them mainly for degradation. These E6-PDZ interactions exhibit extraordinarily different functions in relation to HPV-induced malignancy, depending upon various cellular contexts; for example, Dlg and Scrib show different distribution patterns from what is seen in normal epithelium, both in localization and in amount, and their loss may be a late-stage marker in malignant progression. Recent studies show that interactions with specific forms of the proteins may have oncogenic potential. In addition, it is interesting that PDZ proteins make a contribution to the stabilization of E6 and viral episomal maintenance during the course of HPV life cycle. Various posttranslational modifications also greatly affect their functions. Phosphorylation of hDlg and hScrib by certain kinases regulates several important signaling cascades, and E6-PDZ interactions themselves are regulated through PKA-dependent phosphorylation. Thus these interactions naturally have great potential for both predictive and therapeutic applications, and, with development of screening tools for identifying novel targets of their interactions, comprehensive spatiotemporal analysis is currently underway.

## 1. Introduction

PDZ domain-containing proteins are ubiquitous protein interaction modules and possess multiple functions that are essential to maintain cellular homeostasis. PDZ domain recognition motifs are often encoded by pathogenic viruses, including high-risk HPVs, and through this motif they target various cellular proteins via protein-protein interactions; these HPV-PDZ interactions are in part associated with virally induced cancer progression. However, as this two-way interplay exhibits diverse characteristics and the activities are often combinatorial, depending upon various cellular contexts, extensive understanding of their roles in diverse aspects of cancer has a long way to go. In this paper we focus on the multiple functions that HPV E6-PDZ interactions potentially have in relation to HPV-induced malignancy, depending upon their localizations, stages along the time-axis of malignant progression, and various posttranslational modifications such as phosphorylation by various kinases and we also consider their potential for both predictive and therapeutic applications in HPV-induced malignancy.

## 2. HPVs as the Cause of Cervical Cancer

When exposed to infectious viruses, humans may eliminate infection by their innate immune system, or the infection may progress in stages from mild symptoms to serious, long-term impairment. Some human viruses have been found to cause cancer, accounting for 10–15% of human cancers worldwide [1], and specific viral oncogenes, such as Tax in human T-cell leukemia virus, are identified as responsible for causing tumors in the infected cells. Perhaps the best studied among them are the E6 and E7 oncoproteins in HPVs, the cause of cervical cancer.

### 2.1. Characteristics of Cervical Cancer

Cervical cancer is the third most common female cancer, with 530,000 new cases and 275,000 deaths in a year [2], and is associated with a subset of high-risk HPVs. The most prevalent of these, types 16 and 18, together account for more than 70% of cases [3]. HPV infections are very common among young sexually active women, and the majority of infections are transient and cleared by their immune system within a short period, with 70% of infections deleted in 1 year and 90% in 2 years [4]. In 10% of women, however, HPVs evade the immune system, resulting in long-term persistent infection, and can cause dysplasia, the precancerous but reversible changes which may slowly progress to cancer [5].

### 2.2. Role of E6 and E7 Oncoproteins in Malignancy

The key feature of HPV-induced malignancy is the sustained high-level expression of E6 and E7, which are encoded by early genes of HPVs. Previous studies have provided abundant evidence of a direct role for E6 and E7 oncoproteins in both the development and maintenance of the malignant state [6], and it has been established, albeit *in vitro*, that the removal of either E6 or E7 from cells derived from cervical tumors results in growth arrest and apoptosis, highlighting their potential for novel diagnostic and therapeutic targets in cervical cancer [7]. However, the long latency period between HPV infection and the manifestation of cervical cancer suggests the recruitment of additional cellular factors in this period.

Mechanisms underlying the abilities of both proteins to induce malignancy are diverse and complex. Certainly, the ability of E7 to override cell cycle regulation (stimulate S-phase progression) by interaction with the pocket protein family, including the retinoblastoma tumor suppressor (pRb) is critical [8], together with the cooperating ability of E6 to counteract apoptotic pathways induced as a natural response to the unscheduled S-phase entry mediated by E7 [9]. Indeed, E6 is a multifunctional protein, and a large number of cellular proteins have been found to interact with it; among the most well-known binding partners is p53, the tumor suppressor, which it targets for proteasome-mediated degradation [10]. However, E6's oncogenic activities cannot be explained solely by its effects on p53, and mutants deficient for degradation of p53 can still immortalize mammary epithelial cells, suggesting that interactions with cellular factors other than p53 are necessary for cancer development [11]. One class of cellular proteins that has emerged to fit this role is the PDZ (PSD-95/Dlg/ZO-1) domain-containing substrates of E6. Importantly, transgenic mice encoding E6 proteins defective for binding to PDZ domains do not develop hyperplasia or tumors [12], indicating the importance of this interaction for viral malignant transforming activity.

## 3. HPV E6 and Its PDZ Domain-Containing Targets

### 3.1. Functions of PDZ Domain-Containing Proteins and Their Interactions through PBM

PDZ domains are ubiquitous protein interaction modules conserved throughout evolution [13] and are mainly found in cytoplasmic and membrane adaptor proteins [14]. They are involved in numerous activities within the cells, including regulation of cell polarity, cell proliferation, cell migration and invasion, cell attachment and cell-cell contact, apoptosis and immune cell recognition, and signal transduction pathways [15], the perturbations of which are key characteristics of epithelial cancers. The PDZ domain generally consists of approximately 80–90 amino acid residues that act as modules and scaffolds for protein interactions, and this domain has a structurally well-defined interaction “pocket” which can be filled with a PDZ-binding motif “ligand”. The PDZ-binding motif (PBM) is a specific sequence, often located at the extreme carboxyl terminus, and PDZ domains can interact with a multitude of proteins that possess PBMs. Previous studies in *Drosophila* have shown that some PDZ domain-containing substrates regulate cell growth and polarity, and loss of these substrates results in excessive overproliferation and neoplastic transformation of epithelial cells, thereby defining this type of PDZ domain-containing proteins as potential tumor suppressors [16]. However, in higher eukaryotes, the roles of these proteins are less known, and their functions are more various and complex.

**Figure 1:**
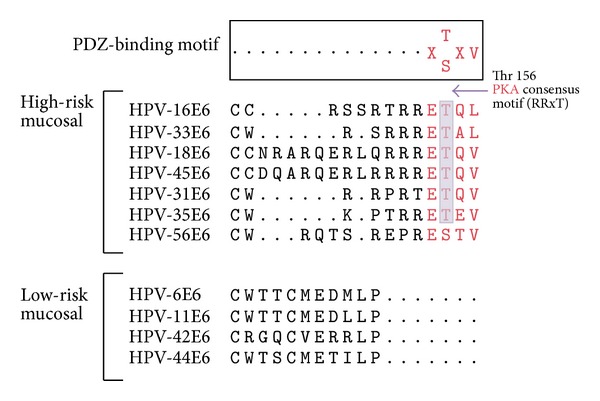
The E6 proteins of high-risk or oncogenic HPVs uniquely contain class I -PBM (sequence x-S/T-x-V/I/L-COOH) located at the C-terminus. Representative alignment of the C-terminal ends of the E6 proteins from the most frequently found. HPV types associated with cervical carcinoma (HPV 16, 33, 18, 45, 31, 35, 56) and compared with low risk types (HPV 6, 11, 42, 44). PKA consensus motif (RRxT) marked in purple.

Interestingly, the E6 proteins of high-risk or oncogenic HPVs uniquely contain a class I -PBM (sequence x-S/T-x-V/I/L-COOH) located at the C-terminus, and this is absent in those HPV E6 proteins that are only associated with benign disease, a fact suggestive of the PBM being a distinctive signature for malignant potential among HPVs [17, 18] (Figure 1). Through this PBM, E6 interacts with a variety of cellular proteins that possess PDZ domains, the majority of which are associated with the regulation of cell polarity, and targets those PDZ-containing proteins for proteasome-mediated degradation [19].

### 3.2. HPV E6 and Its Multiple Substrates: The Interactions Greatly Affect Both Malignant Progression and Viral Life Cycle

Many potential PDZ-containing proteins targeted by E6 have been identified and intensively studied, including hDlg1 (a human homologue of *Drosophila* discs large 1) [17, 18], hScrib (a human homologue of *Drosophila* Scribble) [20], MAGI-1 (membrane-associated guanylate kinase with an inverted arrangement of protein-protein interaction domains), [21] and many others. Both hDlg1 and hScrib are core components of polarity control at adherens junctions, and MAGI-1 is essential for tight junction integrity. It is worth noting that not all the high-risk HPV E6 proteins recognize their different PDZ domain-containing substrates with equal affinity, and minor alterations in the PBM of E6 greatly alter substrate selection. For example, hScrib is preferentially targeted by HPV-16 E6, whilst hDlg is targeted by HPV-18 E6 [22]. Whether these differences will ultimately be reflected in different disease pathologies associated with different virus types still remains to be seen, but it seems to be an intriguing possibility.

In addition to its contribution to the E6-related acquisition of malignant transforming characteristics, the PDZ-PBM interaction has a crucial role in the HPV life cycle. Studies in human foreskin keratinocyte (HFK) cell lines transfected with HPV-31 genomes have demonstrated that mutant viruses defective in PDZ binding lead to reduced growth rates, loss of viral episomes, and increased numbers of unstable viral genomes that are either lost or become integrated into the host chromosomes [23]. Considering the fact that modulation takes place in the earlier stages of HPV life cycle, that is, in the viral proliferation and maintenance phase rather than later in malignant progression, it is reasonable to speculate that E6 PBM-PDZ interactions occur in a variety of cellular contexts and at different stages along the time axis of malignant progression.

In the discussion below, we focus on what effect these varieties of HPV E6-PDZ interactions potentially have in relation to HPV-induced malignancy, depending upon “where” (different localization) and “when” (different stages in viral life cycle or in cancer progression) they take place and upon various posttranslational modifications to which they are subject.

## 4. HPV-PDZ Interplay during the Course of HPV Life Cycle

### 4.1. A Model for Progression from HPV Infection towards Malignancy

**Figure 2:**
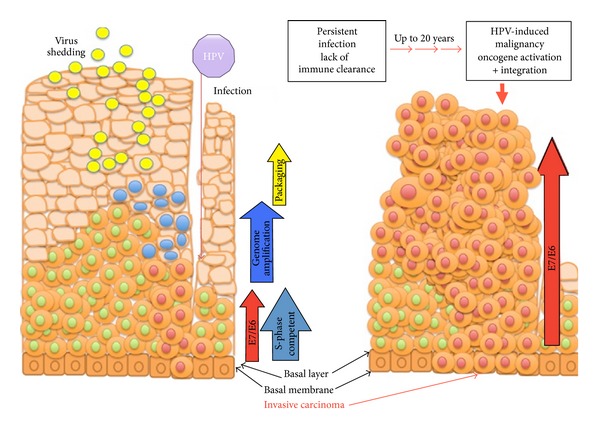
The existing model for the progression from HPV infection towards malignancy. HPV infection occurs when microtraumas allow HPVs to enter the mucosal basal cell layer of genital tract epithelium. Initially, HPVs maintain the genome at low copy number as episomes in the basal cells of epithelium and thereby are capable of establishing long-term latent infections. However, when HPVs succeed in evading innate immune recognition and elimination, continued persistence of the viruses can cause dysplasia, the precancerous but reversible change, which may slowly progress to cancer.

The existing model for the progression from HPV infection towards malignancy is shown in Figure 2 [24, 25]. Initial HPV infection occurs when microtraumas secondary to sexual intercourse allow HPVs to enter the mucosal basal cell layer of genital tract epithelium. Initially, HPVs maintain their genome at low copy number, around 10–200 copies per cell, as episomes in the basal cells of the epithelium, and thereby are capable of establishing long-term latent infections. For viral replication, viruses depend on the terminal differentiation of stratified epithelium and are known to alter the host's differentiation program to allow it to reenter cell cycle through the coordinate expression of viral gene products including E6 and E7. This process, known as the proliferative phase, is subsequently followed by genome amplification, virus synthesis, and shedding of new viral particles within a short period of 2-3 weeks postinfection. As previously mentioned, when considering high prevalence of HPV infections in the young sexually active population, the number of lesions that ultimately progress to cancer is fairly low that is, the majority of infections are transient and cleared by the immune system. However, when HPVs succeed in evading innate immune recognition and elimination, continued persistence of the viruses can cause dysplasia, the precancerous but reversible changes that may slowly progress to cancer.

### 4.2. Stabilization of HPV16 E6 Protein by PDZ Proteins

Studies on NIKS cells, a spontaneously immortalized human keratinocyte cell line that can support the entire life cycle of HPV-16 [26], have shown that the presence of hScrib significantly increased the levels of E6 protein, revealing that the interactions between E6 PBM and its PDZ-containing targets may increase the stability of E6 during early stages of the viral life cycle [27]. Furthermore, mutant HPV-16 genomes lacking E6 PBM cannot persist in an episomal state, and this instability leads either to their being rapidly degraded by the proteasome or being integrated into the host cellular DNA. In addition to hScrib, other PDZ domain-containing proteins, including hDlg1 and MAGI-1, have also been suggested to stabilize E6 expression in a PBM-dependent manner.

This is consistent with the previous study on HFK cells transfected with HPV-31, where mutation of PBM resulted in significant retardation in their growth rates and reduction in their viral copy number, together with the unusual morphological differentiation of cells [23]. 

It is somewhat unexpected, but appears to be a curious paradox, that the same series of proteins which E6 targets for degradation during malignant progression fulfill an important role for the stabilization of E6 and the episomal maintenance in earlier stages of the HPV life cycle, suggesting the possibility of the multiple functions for PDZ-PBM interactions, depending upon different cellular contexts.

## 5. HPV-PDZ Interplay during Malignant Progression

### 5.1. Perturbation of the Functions of PDZ Proteins Results in Major Characteristics of Various Cancers

As definitively discussed in previous studies, both hDlg and hScrib are intimately linked to homeostatic maintenance of several epithelial tissues, including regulation of cell polarity and proliferation. Deregulation of these proteins has been shown in a number of human epithelial cancers: mislocalized and deregulated hScrib expression is seen in colorectal [28], breast [29], prostate [30], and endometrial cancers [31], providing further powerful evidence supporting a role for these proteins in tumor suppression. As for cervical cancer, hDlg and hScrib are degraded to varying degrees by high-risk HPV E6 oncoproteins, both in cells derived from cervical tumors and in a variety of experimental settings, and their expression levels are low in late-stage cancer [32]. Thus, loss of these regulatory functions appears to be a major feature in diverse malignancies.

### 5.2. Spatiotemporal Regulation of PDZ Proteins—Their Loss Might Be a Late-Stage Marker in Cervical Cancer

Interestingly, changes in the levels of expression and localization of Dlg and Scrib occur dependently upon different stages of malignant progression to cervical cancer. For Dlg, analysis by immunohistochemistry showed that the expression of Dlg in cervical intraepithelial precursor lesions displays a distribution pattern different from what is seen in normal epithelium, that is, an unusual increase in the cytoplasm and loss at the sites of cell-cell contact (normal localization). It was only in the invasive form of cervical cancers that a severe decrease in the amount was observed [33], suggesting that loss of Dlg is a late-stage marker in cervical malignancy but that alterations in its normal expression and localization may also contribute to the progression from precancerous lesions to cervical cancer. Also, it has been demonstrated that for proteasome-mediated degradation, E6 preferentially targets nuclear pools of hDlg rather than membrane-bound forms, which shows an example of a localization-dependent difference in susceptibility of PDZ-containing protein to E6 [34].

In addition, the analysis of Scrib showed redistribution during cervical carcinogenesis, from cell contact sites in the squamous cells towards the cytoplasm in dysplastic cells, with a gradual reduction in the Scrib expression levels along with the tumor progression [35].

### 5.3. HPV-PDZ Interaction Could Have Oncogenic Potential in Certain Context

A recent study, showing that SGEF (Src homology 3 domain-containing guanine nucleotide exchange factor) is a strong binding partner of Dlg and that the interaction of the two induces activation of RhoG both *in vitro* and *in vivo*, has revealed that specific forms of Dlg, which remain bound by E6 but not degraded, may actually have oncogenic potential [36], which is another novel aspect of E6 and its PDZ-containing interaction. 

Thus, PDZ domain-containing proteins are implicated in diverse aspects of tumor growth, development, and metastasis.

## 6. Changes in Cell Signaling by Posttranslational Modifications Also Greatly Influence the Interplay between E6 and Its PDZ-Containing Targets

**Figure 3:**
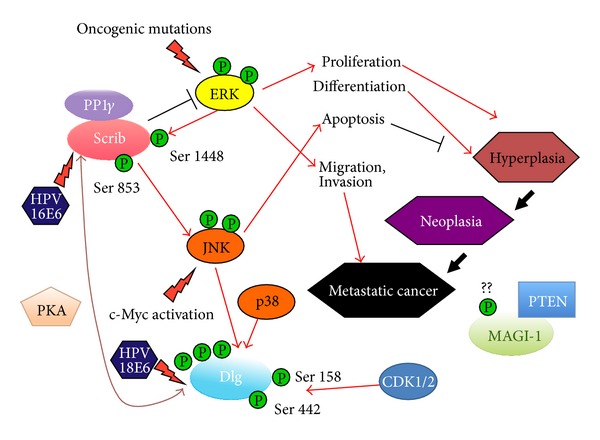
Multifunctional phosphorylated PDZ proteins integrate signaling pathways to control hyperplasia, neoplasia, and cell metastasis. Modified forms of PDZ proteins are potential molecular markers predicting the aggressiveness of malignancy. Candidates include Dlg phosphorylated by PKA, JNK, CDK1, or CDK2; Scrib interaction with ERK; Scrib recruitment of PP1gamma for downregulation of the MAPK pathway. No studies that could offer an explanation of the phosphorylation of MAGI-1 and PTEN by MAPK are currently available.

In addition to the two distinct aspects of HPV E6-PDZ interactions at different stages along the time axis of HPV-induced malignant progression, as mentioned above, recent studies have shown that these PDZ domain interactions can be modulated by a multitude of posttranslational modifications, the most important of which is phosphorylation. Indeed, specific phosphorylation states have been shown to greatly affect the localization and the susceptibility of each PDZ-containing protein to proteasome-mediated degradation by E6 (Figure 3).

### 6.1. Phosphorylation of hDlg by JNK, CDK1, and CDK2

For instance, hyperphosphorylation of hDlg by Jun N-terminal kinase (JNK), which occurs in response to osmotic shock, results in the rapid accumulation of Dlg at the sites of cell contact and renders it more susceptible to degradation induced by HPV-18 E6 [37]. Another example of the phosphorylation-dependent regulation of hDlg is mediated by two major cell cycle regulatory kinases, the cyclin-dependent kinases 1 and 2 (CDK1 and CDK2). When hyperphosphorylated on the specific phosphoacceptor sites of Ser 158 and Ser 442 by these two kinases, Dlg shows a preferential nuclear accumulation and thereby enhanced susceptibility to E6-induced degradation, implicating its nuclear function as a tumor suppressor in the late-stage progression of cervical cancer [38]. It is also important to note that these phosphoacceptor sites can also be phosphorylated by mitogen-activated protein kinase (MAPK) in response to the environmental change of osmotic stress, shown by the previous study [39].

### 6.2. hScrib and ERK/MAPK Cascade

The ERK/MAPK cascade, another notable cell signaling pathway, has been reported to be activated in cervical cancers [40], and hScrib is also a substrate of ERK [41]. In human skin keratinocytes, the interaction between hScrib and ERK, through hScrib's recruitment of a protein phosphatase, PP1gamma, for the dephosphorylation of ERK, subsequently inactivates ERK and inhibits its nuclear translocation, ultimately resulting in the downregulation of the MAPK signaling cascade [42]. Whether this interaction between hScrib and ERK actually happens in the progression of cervical cancer deserves further investigation, but it is highly likely that the downregulation of ERK/MAPK cascade is one of hScrib's multifunctional activities as tumor suppressor (Figure 3).

### 6.3. Regulation of HPV-PDZ Interactions through PKA-Dependent Phosphorylation

Interestingly, not only are the PDZ-containing proteins regulated through phosphorylation, but so also is HPV E6. Embedded within the HPV E6 PBM there is a consensus recognition site for protein kinase A (PKA) phosphorylation on the Thr/Ser residue at the −3 position (Figure 1). Previous studies showed that both PKA- and PKC-dependent phosphorylation can lead to the inhibition of many ligand-PDZ interactions ([43, 44] and more), and this would also seem to be true for HPV-18 E6 *in vivo*. Thus, the E6-PBM phosphorylation by PKA greatly inhibits E6-Dlg recognition and subsequent degradation, thereby maintaining high Dlg protein levels under conditions of high PKA activity [45]. A striking feature of this PKA recognition motif is its strict conservation among the high-risk HPV E6 proteins and its absence in the low-risk HPV E6 proteins, suggesting that PKA-regulated degradation of hDlg is an essential function in the process of malignancy [46]. However, the PKA recognition motif within the E6-PBM not only has a role in regulating PDZ-binding activity, but a recent study shows that the phosphorylation of E6 by PKA or AKT is also significant for the interaction between certain types of HPV E6 and 14-3-3*ζ* and that this E6/14-3-3*ζ* interaction is essential to maintain E6 levels [46]. Thus, this finding suggests that, in addition to the endogenous PKA activity, the expression level of 14-3-3*ζ* in high-risk HPV positive cervical tissues could be considered as a potential biomarker for predicting the development of cervical cancer.

At present, no information is available about at which stage, either during malignancy or during the course of viral life cycle, and to what degree, this E6 phosphorylation by PKA or AKT takes place, but this feature assumes great importance for the prospective designs for both progression-risk prediction and treatment for cervical cancer, considering the correspondent fact shown by many previous studies that mutations that disrupt normal PKA activity are frequently found in many forms of malignancy.

Thus, modifications by kinases and changes in cellular signaling pathways can profoundly influence, and sometimes dramatically alter, the efficiency of E6-PDZ interplay.

## 7. Other Possible Functions of PDZ-Containing Proteins in relation to the Development of Cervical Cancer

### 7.1. Immune Response

There are some fascinating recent studies that indicate PDZ targeting by E6 might be related to viral evasion of the host innate immune response. The important virulence factor of the Influenza A virus, NS 1 (nonstructural protein 1), has no oncogenic potential but does have a PBM at the carboxyl terminus and binds to PDZ-containing proteins as HPV E6 does [47]. The importance of the PBM for Influenza virus virulence has been associated with impairment of interferon activity via the downregulation of JAK/STAT signaling pathway [48]. Thus a possible analogy is that E6, through targeting certain PDZ substrates, can weaken the innate immune response. Indeed, previous studies have shown that E6 can impair JAK/STAT signaling [49], although whether E6-PDZ domain-containing substrates play a role in this activity remains to be determined.

### 7.2. Chronic Inflammation

The relationship between cancer progression and chronic inflammation has long been reported, and a transcription factor, nuclear factor-kappa B (NF*κ*B), is well known as a driving force for generating chronic inflammation in the pathogenesis of many cancers, in addition to being implicated in a variety of other processes including proliferation, migration, angiogenesis, and prevention of apoptosis. Toll-like receptors (TLR) are possible signal initiators for NF*κ*B activation, due to inflammation-inducing carcinogenesis, and are also known to be the forefront receptors in response to microbial infection. Therefore, activation of NF*κ*B through stimulated TLR in local chronic inflammation may serve as an initiator of carcinogenesis [50]. Previous studies have reported the activation and subsequent overexpression of NF*κ*B in HPV-related cancers, and again the PBM of E6 appears to be required for this process [51].

### 7.3. Epithelial-Mesenchymal Transition

Furthermore, it was shown that in keratinocytes the PBM is also important for both HPV-16 and HPV-18 E6 to promote morphological changes that eventually lead to epithelial-mesenchymal transition (EMT), another crucial hallmark of cancer progression, especially in the progression of metastasis [52]. Therefore it is natural to infer that loss of PDZ-containing substrates of E6, such as Dlg1, Scrib, and MAGI-1, might contribute to the promotion of EMT, thereby enhancing the metastatic behavior of the virally transformed cells. A recent intensive study in human keratinocytes shows that an important consequence of Scrib-depletion in these cells is a significant increase in the invasive potential; in contrast, Dlg1-depletion in the same cells has distinct and opposite effects [53]. Also, the observations demonstrate that loss of Scrib can enhance cell invasiveness without the presence of extra oncogenes, such as *myc* and *ras*. In contrast, loss of Dlg-1 can result in the increased resistance of the cells to anoikis. Although the mechanism by which invading tumor cells survive the anoikis process remains largely unknown, we now know that the situation is rather more complex and that loss of Scrib and Dlg-1, which is often seen in malignant tumors, cooperatively contributes to the malignant state. Thus, the study demonstrating the opposing function of these polarity proteins may shed a light on their critical roles in regulating the promotion of EMT. Obviously, future studies are warranted to clarify how these epithelial cell polarity proteins may contribute to tumor suppression. 

## 8. What Can We Learn from These HPV-PDZ Interactions for Cervical Cancer Prevention and Treatment?

Whilst previous studies have revealed much of the requirement for the oncoproteins of E6 and E7 in the development and maintenance of cervical cancer, much still merits further investigation to precisely understand the functions of these two viral proteins. In this review, we have attempted to feature one particular aspect of E6 function that may help to provide some clues to the molecular mechanisms that underlie HPV-induced development of cancer. Now the subject we must focus on is the prospective application of PBM-PDZ interactions for both the prevention and treatment of cervical cancer.

### 8.1. Possible Candidates for Molecular Markers Predicting Both Malignant Progression and Its Aggressiveness

Focusing on diagnostic tools, there is an urgent demand for the development of new biomarkers that can discriminate lesions with a high risk of progression towards cancer from those that will spontaneously regress. As noted above, most infections even with the high-risk HPVs occur without any clinical symptoms, are eliminated by the host immune system within the short period, and do not progress to cancer. High-grade cervical intraepithelial neoplasia (CIN) is normally the subject of therapeutic intervention, involving either removal or destruction of the lesions, to prevent malignant progression, and while this intervention certainly reduces the cancer development rates, the number of patients subject to overmedication increases at the same time. It is fairly reasonable to suggest that a more direct CIN predictor for malignant progression would be desirable. 

Among the PDZ-containing proteins, MAGI-1, hDlg, and hScrib are degraded to varying degrees by high-risk HPV E6 oncoproteins, both in cells derived from cervical tumors and in a variety of experimental settings. However, although Dlg is targeted by E6 for degradation and is lost during the later stages of cervical cancer progression, only certain pools of the Dlg are degraded; therefore substantial amounts of Dlg protein, though often with unusual distribution pattern or phosphorylated forms, are found in HPV-positive tumor cells [34]. As mentioned above, several modified forms of PDZ proteins, for example, specific phosphorylated forms of Dlg (by PKA, JNK, CDK1, and CDK2), direct interaction between Scrib and ERK, or expression levels of PP1gamma recruited by Scrib for the downregulation of MAPK pathway could be candidates both for molecular markers predicting the aggressiveness of malignancy (high growth rate, invasiveness, and metastatic capacity) and for therapeutic targets.

Moreover, one of the hallmarks of cervical cancer progression is the frequent integration of the viral DNA into the host genome, and although there is considerable debate as to the role of this in disease development, the presence of integrated DNA sequences is likely to be of concern due to the concomitant high levels of E6 and E7 gene expression [54]. Therefore one possibility is that loss of PDZ-binding capacity through PKA phosphorylation of E6 might create an environment that is conducive to the loss of viral episomes and to consequent viral integration into the host genome. Whilst this remains speculative, it does nonetheless suggest that the levels of PKA activity within the infected cervical epithelium might be one marker that could be assessed as a means of determining whether viral DNA integration was more or less likely, and studies are currently underway to investigate these aspects further. 

### 8.2. Drug Designs of PDZ-Domain Inhibitors; Possibilities for Novel Potent Therapeutic Intervention

Whether any of these PDZ targets of E6 might have therapeutic potential is also an intriguing possibility. In general, protein-protein interactions provide attractive possibilities for therapeutic intervention, and disruption of specific protein interactions with high affinity is a key concept for producing a molecularly targeted drug. However, targeting protein interactions are clearly more difficult than those targets that naturally bind molecules [55, 56]. Thanks to the advances in technology, we can now identify small molecules that can block specific proteins by screening an extensive small molecule database *in silico*, but the problem is that a large proportion of candidates identified by *in silico* screening prove to be false positive results [57]. Additional methods such as NMR spectroscopy are needed to evaluate these screening results, and indeed some small molecule inhibitors of the PDZ domain have been identified using this novel approach of NMR-assisted virtual screening, and they are shown to potently block specific cell signaling pathways [57, 58]. Thus, this novel *in silico* approach to drug design could be a valuable tool in identifying novel inhibitors of the E6-PDZ interactions, potentially leading to significant advances in the treatment of cervical cancer.

## 9. Conclusions

Although studies are still in their infancy, the HPV-PDZ interactions could have vast potential as both predictive and therapeutic targets in HPV-induced malignancies. And as these proteins appear to be multifunctional, sometimes exerting completely opposite influences on cancer progression depending on contexts, a comprehensive analysis of the PDZ protein expression at different stages along the time axis of malignant progression, and in various posttranslationally modified states, is essential to precisely elucidate the functions of HPV-PDZ interactions, before novel molecular biomarkers or drugs targeting these interactions come into practical use.

## References

[1] Moore PS, Chang Y (2010). Why do viruses cause cancer? Highlights of the first century of human tumour virology. *Nature Reviews Cancer*.

[2] Arbyn M, Castellsagué X, de sanjosé S (2011). Worldwide burden of cervical cancer in 2008. *Annals of Oncology*.

[3] Smith JS, Lindsay L, Hoots B (2007). Human papillomavirus type distribution in invasive cervical cancer and high-grade cervical lesions: a meta-analysis update. *International Journal of Cancer*.

[4] Doorbar J, Quint W, Banks L (2012). The biology and life-cycle of human papillomaviruses. *Vaccine*.

[5] Franco EL, Villa LL, Sobrinho JP (1999). Epidemiology of acquisition and clearance of cervical human papillomavirus infection in women from a high-risk area for cervical cancer. *Journal of Infectious Diseases*.

[6] Münger K (2002). The role of human papillomaviruses in human cancers. *Frontiers in Bioscience*.

[7] Jiang M, Milner J (2002). Selective silencing of viral gene expression in HPV-positive human cervical carcinoma cells treated with siRNA, a primer of RNA interference. *Oncogene*.

[8] Münger K, Basile JR, Duensing S (2001). Biological activities and molecular targets of the human papillomavirus E7 oncoprotein. *Oncogene*.

[9] Mantovani F, Banks L (2001). The human papillomavirus E6 protein and its contribution to malignant progression. *Oncogene*.

[10] Scheffner M, Huibregtse JM, Vierstra RD, Howley PM (1993). The HPV-16 E6 and E6-AP complex functions as a ubiquitin-protein ligase in the ubiquitination of p53. *Cell*.

[11] Liu Y, Chen JJ, Gao Q (1999). Multiple functions of human papillomavirus type 16 E6 contribute to the immortalization of mammary epithelial cells. *Journal of Virology*.

[12] Nguyen ML, Nguyen MM, Lee D, Griep AE, Lambert PF (2003). The PDZ ligand domain of the human papillomavirus type 16 E6 protein is required for E6’s induction of epithelial hyperplasia in vivo. *Journal of Virology*.

[13] Ponting CP (1997). Evidence for PDZ domains in bacteria, yeast, and plants. *Protein Science*.

[14] Javier RT, Rice AP (2011). Emerging theme: cellular PDZ proteins as common targets of pathogenic viruses. *Journal of Virology*.

[15] Subbaiah VK, Kranjec C, Thomas M, Banks L (2011). PDZ domains: the building blocks regulating tumorigenesis. *Biochemical Journal*.

[16] Bilder D (2004). Epithelial polarity and proliferation control: links from the *Drosophila* neoplastictumor suppressors. *Genes and Development*.

[17] Kiyono T, Hiraiwa A, Fujita M, Hayashi Y, Akiyama T, Ishibashi M (1997). Binding of high-risk human papillomavirus E6 oncoproteins to the human homologue of the *Drosophila* discs large tumor suppressor protein. *Proceedings of the National Academy of Sciences of the United States of America*.

[18] Lee SS, Weiss RS, Javier RT (1997). Binding of human virus oncoproteins to hDlg/SAP97, a mammalian homolog of the *Drosophila* discs large tumor suppressor protein. *Proceedings of the National Academy of Sciences of the United States of America*.

[19] Banks L, Pim D, Thomas M (2003). Viruses and the 26S proteasome: hacking into destruction. *Trends in Biochemical Sciences*.

[20] Nakagawa S, Huibregtse JM (2000). Human scribble (Vartul) is targeted for ubiquitin-mediated degradation by the high-risk papillomavirus E6 proteins and the E6AP ubiquitin-protein ligase. *Molecular and Cellular Biology*.

[21] Glaunsinger BA, Lee SS, Thomas M, Banks L, Javier R (2000). Interactions of the PDZ-protein MAGI-1 with adenovirus E4-ORF1 and high-risk papillomavirus E6 oncoproteins. *Oncogene*.

[22] Thomas M, Massimi P, Navarro C, Borg J-P, Banks L (2005). The hScrib/Dlg apico-basal control complex is differentially targeted by HPV-16 and HPV-18 E6 proteins. *Oncogene*.

[23] Lee C, Laimins LA (2004). Role of the PDZ domain-binding motif of the oncoprotein E6 in the pathogenesis of human papillomavirus type 31. *Journal of Virology*.

[24] Doorbar J (2005). The papillomavirus life cycle. *Journal of Clinical Virology*.

[25] Thomas M, Narayan N, Pim D (2008). Human papillomaviruses, cervical cancer and cell polarity. *Oncogene*.

[26] Flores ER, Allen-Hoffmann BL, Lee D, Sattler CA, Lambert PF (1999). Establishment of the human papillomavirus type 16 (HPV-16) life cycle in an immortalized human foreskin keratinocyte cell line. *Virology*.

[27] Nicolaides L, Davy C, Raj K, Kranjec C, Banks L, Doorbar J (2011). Stabilization of HPV16 E6 protein by PDZ proteins, and potential implications for genome maintenance. *Virology*.

[28] Kamei Y, Kito K, Takeuchi T (2007). Human scribble accumulates in colorectal neoplasia in association with an altered distribution of *β*-catenin. *Human Pathology*.

[29] Zhan L, Rosenberg A, Bergami KC (2008). Deregulation of scribble promotes mammary tumorigenesis and reveals a role for cell polarity in carcinoma. *Cell*.

[30] Pearson HB, Perez-Mancera PA, Dow LE (2011). SCRIB expression is deregulated in human prostate cancer, and its deficiency in mice promotes prostate neoplasia. *Journal of Clinical Investigation*.

[31] Ouyang Z, Zhan W, Dan L (2010). hScrib, a human homolog of *Drosophila* neoplastic tumor suppressor, is involved in the progress of endometrial cancer. *Oncology Research*.

[32] Banks L, Pim D, Thomas M (2012). Human tumour viruses and the deregulation of cell polarity in cancer. *Nature Reviews Cancer*.

[33] Cavatorta AL, Fumero G, Chouhy D (2004). Differential expression of the human homologue of *Drosophila* discs large oncosuppressor in histologic samples from human papilloma virus-associated lesions as a marker for progression to malignancy. *International Journal of Cancer*.

[34] Massimi P, Gammoh N, Thomas M, Banks L (2004). HPV E6 specifically targets different cellular pools of its PDZ domain-containing tumour suppressor substrates for proteasome-mediated degradation. *Oncogene*.

[35] Nakagawa S, Yano T, Nakagawa K (2004). Analysis of the expression and localisation of a LAP protein, human scribble, in the normal and neoplastic epithelium of uterine cervix. *British Journal of Cancer*.

[36] Krishna Subbaiah V, Massimi P, Boon SS (2012). The invasive capacity of HPV transformed cells requires the hDlg-dependent enhancement of SGEF/RhoG activity. *PLOS Pathogens*.

[37] Massimi P, Narayan N, Cuenda A, Banks L (2006). Phosphorylation of the discs large tumour suppressor protein controls its membrane localisation and enhances its susceptibility to HPV E6-induced degradation. *Oncogene*.

[38] Narayan N, Massimi P, Banks L (2009). CDK phosphorylation of the discs large tumour suppressor controls its localisation and stability. *Journal of Cell Science*.

[39] Sabio G, Arthur JSC, Kuma Y (2005). p38*γ* regulates the localisation of SAP97 in the cytoskeleton by modulating its interaction with GKAP. *The EMBO Journal*.

[40] Branca M, Ciotti M, Santini D (2004). Activation of the ERK/MAP kinase pathway in cervical intraepithelial neoplasia is related to grade of the lesion but not to high-risk human papillomavirus, virus clearance, or prognosis in cervical cancer. *American Journal of Clinical Pathology*.

[41] Nagasaka K, Pim D, Massimi P (2010). The cell polarity regulator hScrib controls ERK activation through a KIM site-dependent interaction. *Oncogene*.

[42] Nagasaka K, Seiki T, Yamashita A (2013). A novel interaction between hScrib and PP1*γ* downregulates ERK signaling and suppresses oncogene-induced cell transformation. *PLoS One*.

[43] Xia J, Chung HJ, Wihler C, Huganir RL, Linden DJ (2000). Cerebellar long-term depression requires PKC-regulated interactions between GluR2/3 and PDZ domain-containing proteins. *Neuron*.

[44] Sonoda T, Mochizuki C, Yamashita T (2006). Binding of glutamate receptor *δ*2 to its scaffold protein, Delphilin, is regulated by PKA. *Biochemical and Biophysical Research Communications*.

[45] Kühne C, Gardiol D, Guarnaccia C, Amenitsch H, Banks L (2000). Differential regulation of human papillomavirus E6 by protein kinase A: conditional degradation of human discs large protein by oncogenic E6. *Oncogene*.

[46] Boon SS, Banks L (2013). High-risk human papillomavirus E6 oncoproteins interact with 14-3-3*ζ* in a PDZ binding motif-dependent manner. *J Virol*.

[47] Jackson D, Hossain MJ, Hickman D, Perez DR, Lamb RA (2008). A new influenza virus virulence determinant: the NS1 protein four C-terminal residues modulate pathogenicity. *Proceedings of the National Academy of Sciences of the United States of America*.

[48] Thomas M, Kranjec C, Nagasaka K, Matlashewski G, Banks L (2011). Analysis of the PDZ binding specificities of Influenza A Virus NS1 proteins. *Virology Journal*.

[49] Li S, Labrecque S, Gauzzi MC (1999). The human papilloma virus (HPV)-18 E6 oncoprotein physically associates with Tyk2 and impairs Jak-STAT activation by interferon-*α*. *Oncogene*.

[50] Senba M, Mori N (2012). Mechanisms of virus immune evasion lead to development from chronic inflammation to cancer formation associated with human papillomavirus infection. *Oncology Reviews*.

[51] James MA, Lee JH, Klingelhutz AJ (2006). Human papillomavirus type 16 E6 activates NF-*κ*B, induces cIAP-2 expression, and protects against apoptosis in a PDZ binding motif-dependent manner. *Journal of Virology*.

[52] Watson RA, Thomas M, Banks L, Roberts S (2003). Activity of the human papillomavirus E6 PDZ-binding motif correlates with an enhanced morphological transformation of immortalized human keratinocytes. *Journal of Cell Science*.

[53] Massimi P, Zori P, Roberts S, Banks L (2012). Differential regulation of cell-cell contact, invasion and anoikis by hScrib and hDlg in keratinocytes. *PLoS One*.

[54] Jeon S, Lambert PF (1995). Integration of human papillomavirus type 16 DNA into the human genome leads to increased stability of E6 and E7 mRNAs: implications for cervical carcinogenesis. *Proceedings of the National Academy of Sciences of the United States of America*.

[55] Arkin MMR, Wells JA (2004). Small-molecule inhibitors of protein-protein interactions: progressing towards the dream. *Nature Reviews Drug Discovery*.

[56] Dev KK (2004). Making protein interactions druggable: targeting PDZ domains. *Nature Reviews Drug Discovery*.

[57] Grandy D, Shan J, Zhang X (2009). Discovery and characterization of a small molecule inhibitor of the PDZ domain of dishevelled. *Journal of Biological Chemistry*.

[58] Shan J, Zhang X, Bao J, Cassell R, Zheng JJ (2012). Synthesis of potent dishevelled PDZ domain inhibitors guided by virtual screening and NMR studies. *Chemical Biology and Drug Design*.

